# Non-operative Approach to an Incidental Internal Jugular Venous Aneurysm: A Case Report

**DOI:** 10.7759/cureus.111580

**Published:** 2026-06-26

**Authors:** Erik Gamarra, Zachary Fent, Maria Miglio, Mazen Bazzi, Alexander A Restum

**Affiliations:** 1 General Surgery, Corewell Health Dearborn Hospital, Dearborn, USA; 2 Vascular Surgery, Corewell Health Dearborn Hospital, Dearborn, USA; 3 Medical Education, Wayne State University School of Medicine, Detroit, USA

**Keywords:** internal jugular venous aneurysm, neurofibromatosis 1, vascular malformations, vascular surgery, venous aneurysms

## Abstract

Internal jugular venous aneurysms (IJVAs) are rare vascular malformations that usually manifest as soft, pulsatile neck masses that are often misdiagnosed. In this case, we present a 70-year-old man with fatigue, dyspnea, and an intermittent pulsating neck sensation in the setting of acute anemia. Computed tomography angiography (CTA) revealed a 2.7 cm saccular aneurysm of the left internal jugular vein with no thrombus. Normally, these aneurysms are asymptomatic; however, they may be surgically remediable on a case-by-case basis, depending on the presence of symptoms, aneurysm morphology, fragility of the vessel wall, and complication risk. Given our patient’s resolving symptoms with correction of anemia, absence of residual paresthesias, and overall surgical risk, conservative management was chosen. There is little literature on IJVAs, particularly in the context of management in the elderly with co-morbid illnesses. This report stresses the need for personalized treatment modalities and contributes to the literature regarding the management options of this rare vascular entity.

## Introduction

Venous aneurysms are vascular malformations characterized by dilatation of a vein. Compared to arterial aneurysms, venous aneurysms normally remain undiagnosed and are asymptomatic [1]. While extremely uncommon, they have been discovered in all veins in the human body, most commonly the popliteal vein [2]. It is reported that 77% of all venous aneurysms occur in the lower extremities and 10% in the upper extremities [3]. Of the venous aneurysms, internal jugular venous aneurysms (IJVAs) amount to a fairly uncommon subgroup, and more often, are identified in younger patients or in those with genetic conditions such as neurofibromatosis 1, alternatively known as Recklinghausen disease [4,5]. IJVAs often present with a soft, pulsating, and compressible mass in the neck and are diagnosed on computed tomography (CT) scan or ultrasound (US) imaging studies. Due to their rarity and non-specific presentation, IJVAs are most likely to be misdiagnosed as cysts, lymphomas, lipomas, or other pathologies of the head and neck. The management of each IJVA is individualized to patient symptoms, aneurysm morphology, fragility of the vessel wall, cosmetic considerations, and risk of perioperative complications. While there are a variety of surgical approaches, ligation and excision are recommended for symptomatic or high-risk cases and result in a greater likelihood of favorable outcomes [6]. In this report, we outline the case of an incidentally identified IJVA diagnosed in a 70-year-old man who presented to the hospital with a pulsating neck mass, fatigue, and shortness of breath. Next, we discuss the imaging modalities, diagnostic approach, and provide a demonstration of successful non-operative management of this uncommon vascular anomaly.

## Case presentation

We present the case of a 70-year-old man who presented to the hospital for evaluation of fatigue and shortness of breath. Significant medical history included coronary artery disease, hypertension, diabetes mellitus, and chronic lymphocytic leukemia (CLL) treated with a chemotherapy regimen and in remission since 2015. In the emergency department, the patient complained of chest tightness that worsened with activity, for which a cardiac workup was negative. He also reported experiencing a cough and feeling an abnormal pulsating sensation in his neck that began three weeks ago. Although no laterality was initially specified, a CT arterial (CTA) of the head and neck was obtained in addition to CTA thorax to rule out pulmonary embolism (PE) as a possible source for his shortness of breath.

**Figure 1:**
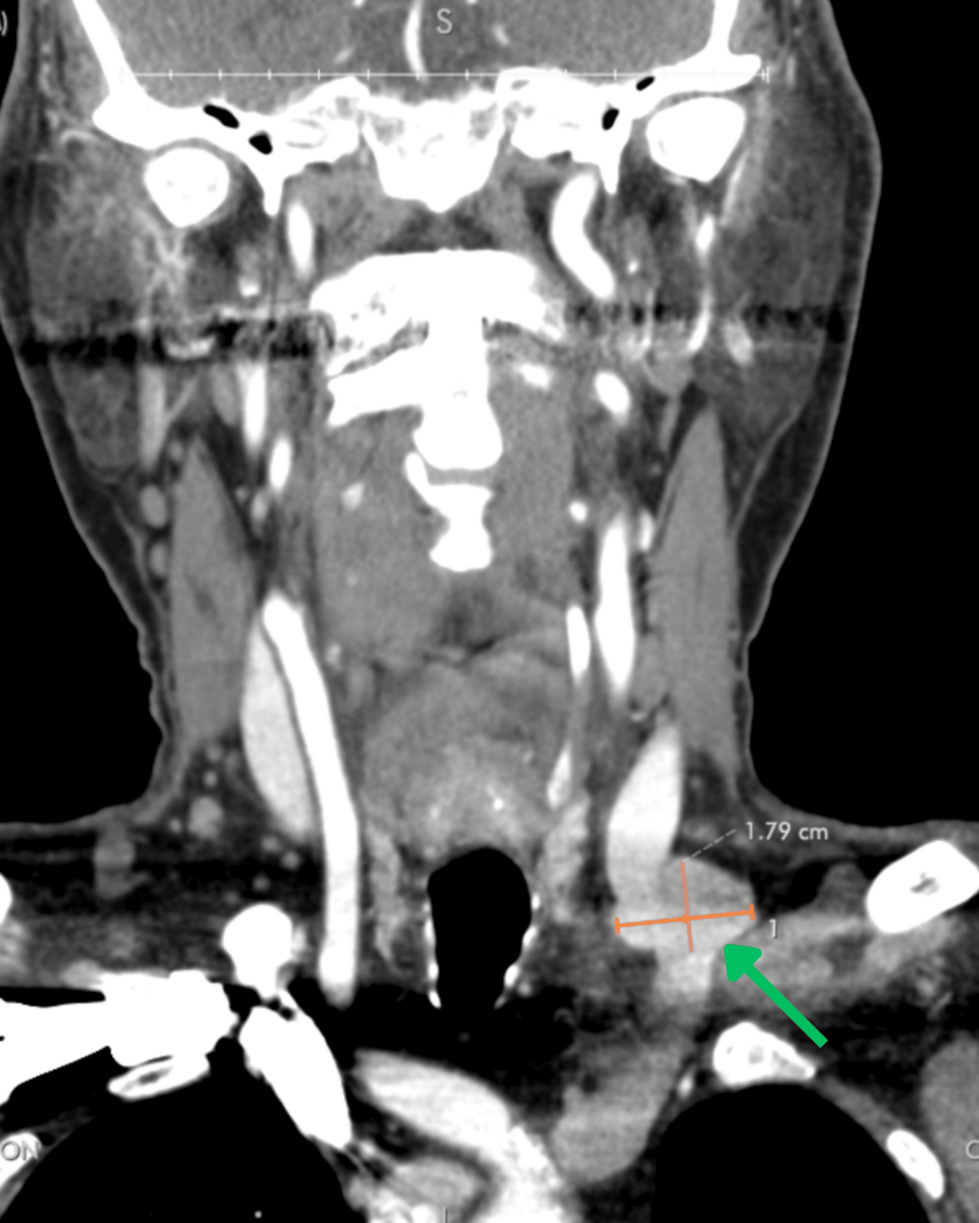
Green arrow pointing to the internal jugular venous aneurysm; orange lines indicate boundaries of the aneurysm.

While no PE was identified, the CTA of the head and neck revealed clinically insignificant atherosclerotic disease of the carotid arteries and a saccular aneurysm at the base of the left internal jugular vein measuring 2.7 cm, as demonstrated in Figure 1. There was no evidence of thrombus within the aneurysm. His symptoms of fatigue and chest tightness improved after laboratory workup revealed anemia with hemoglobin of 5.2 g/dL and platelets of 9,000 × 10^3^/µL, for which appropriate transfusions were administered. Both his anemia and thrombocytopenia were likely attributed to his history of CLL.

Consultation was placed to the vascular surgery team, and the patient described a left neck pulsation upon standing up from a seated position, which resolved within a few seconds. He denied any pain or swelling of the left neck or face, acute sensorimotor deficits, and was no longer feeling lightheaded after receiving blood product transfusions. He had not previously been prescribed antiplatelet or anticoagulation medication, and he did not have a history of trauma or previous vascular access to the left neck. In this case, given his resolution of symptoms following treatment of his anemia, and low likelihood of thrombosis given the morphology of his IJVA, we elected not to proceed with operative intervention. The patient was instructed to follow up in the clinic one month later to monitor his anemia and symptoms. 

## Discussion

IJVAs are rare vascular malformations, accounting for approximately 13% of the total prevalence of venous aneurysms [2]. IJVAs occur in patients across all ages and manifest with a variety of seemingly disjointed clinical presentations. This variability in symptomatology may contribute to missed diagnosis and delayed findings [7], as in our case above of a 70-year-old man with a positionally incited pulsatile neck sensation, ultimately diagnosed with an incidental left IJVA via CTA performed for the evaluation of possible PE. There is limited literature regarding the prevalence and treatment of IJVAs in this population, particularly as most cases described occur in younger patients or those with genetic syndromes such as neurofibromatosis type 1 [8]. Furthermore, the treatment and optimal diagnostic workup for this aneurysm are also contested, with no clear consensus within the literature [9]. Our patient was not known to have a history of trauma, vascular surgery, or genetic disorder - risk factors commonly associated with an IJVA, showing that IJVAs can be idiopathic, especially in elderly patients with coexistent vascular risk factors [6].

While CTA aided in our patient's diagnosis of the IJVA, follow-up Doppler US examination would have been useful for dynamic information. Doppler US is regarded as the initial imaging technique for the evaluation of venous aneurysms, as it enables the calculation of flow dynamics, thrombus detection, wall integrity assessment, and compressibility assessment [1]. For scenarios where thrombus is identified or if aneurysms pose an embolic risk, minimally invasive procedures, such as US-guided thrombin injection, have become safe options compared to open surgery, particularly in anatomically suitable lesions [6]. Given the patient's improving symptoms after anemia correction, absence of chronic neck or face swelling, and significant surgical risk profile, non-surgical management was chosen for treatment; this is a common approach to the management of this condition [10]. This case underscores the need for continued investigation into the condition, including both short- and long-term outcome data, to help guide evidence-based protocols for diagnosis and treatment of IJVAs.

## Conclusions

IJVAs are exceedingly rare circulatory findings due to the fact that the venous system is low-pressure, which typically prevents the development of aneurysms. When such aneurysms occur, they present with a broad range of symptoms, making them difficult to diagnose. This case highlights the importance of considering IJVAs in the differential diagnosis of neck masses and using appropriate imaging modalities to more routinely identify and better characterize these findings. Management of this condition should be patient-tailored, with conservative strategies being favored in asymptomatic patients, and surgical approaches considered for those who are symptomatic or are at higher risk of future complications if left untreated. A greater understanding of IJVA diagnostic criteria and ongoing studies of their progression are necessary to guide future clinical decision-making.
